# The Effect of Maternal Dietary Patterns on Birth Weight for Gestational Age: Findings from the MAMI-MED Cohort

**DOI:** 10.3390/nu15081922

**Published:** 2023-04-16

**Authors:** Martina Barchitta, Roberta Magnano San Lio, Maria Clara La Rosa, Claudia La Mastra, Giuliana Favara, Giuliana Ferrante, Fabiola Galvani, Elisa Pappalardo, Carla Ettore, Giuseppe Ettore, Antonella Agodi, Andrea Maugeri

**Affiliations:** 1 Department of Medical and Surgical Sciences and Advanced Technologies “GF Ingrassia”, University of Catania, 95123 Catania, Italy; 2 Department of Obstetrics and Gynaecology, Azienda di Rilievo Nazionale e di Alta Specializzazione (ARNAS) Garibaldi Nesima, 95124 Catania, Italy

**Keywords:** maternal diet, pregnancy, birth weight, preterm birth, neonatal outcomes, nutrition

## Abstract

Limited evidence exists on the effects of maternal dietary patterns on birth weight, and most studies conducted so far did not adjust their findings for gestational age and sex, leading to potentially biased conclusions. In the present study, we applied a novel method, namely the clustering on principal components, to derive dietary patterns among 667 pregnant women from Catania (Italy) and to evaluate the associations with birth weight for gestational age. We identified two clusters reflecting distinct dietary patterns: the first one was mainly characterized by plant-based foods (e.g., potatoes, cooked and raw vegetables, legumes, soup, fruits, nuts, rice, wholemeal bread), fish and white meat, eggs, butter and margarine, coffee and tea; the second one consisted mainly of junk foods (sweets, dips, salty snacks, and fries), pasta, white bread, milk, vegetable and olive oils. Regarding small gestational age births, the main predictors were employment status and primiparity, but not the adherence to dietary patterns. By contrast, women belonging to cluster 2 had higher odds of large for gestational age (LGA) births than those belonging to cluster 1 (OR = 2.213; 95%CI = 1.047–4.679; *p* = 0.038). Moreover, the odds of LGA increased by nearly 11% for each one-unit increase in pregestational BMI (OR = 1.107; 95%CI = 1.053–1.163; *p* < 0.001). To our knowledge, the present study is the first to highlight a relationship between adherence to an unhealthy dietary pattern and the likelihood of giving birth to a LGA newborn. This evidence adds to the current knowledge about the effects of diet on birth weight, which, however, remains limited and controversial.

## 1. Introduction

Globally, about 140 million births take place every year. Deaths from complications during pregnancy and childbirth have declined by 38% in the last two decades, but at an average reduction of just 3% per year [1]. Among maternal complications, gestational diabetes mellitus and inadequate gestational weight gain (GWG) constitute two of the main threats for maternal-child health [2,3,4,5,6,7]. Similarly, several adverse neonatal outcomes, including pre-term birth (PTB), low birth weight (LBW) and macrosomia, small or large for gestational age (SGA and LGA) birth, and intrauterine growth restriction (IUGR) continue to represent major public health problems [8,9,10,11,12,13]. In general, both prematurity and LBW remain the leading causes of deaths among newborns and children. Their consequences may manifest immediately—leading to a higher risk of developmental disabilities (e.g., cerebral palsy and retinopathy)—or in adulthood. It is, in fact, well established that LBW infants also have a higher risk of adult chronic conditions, including obesity and diabetes. Similarly, infants who are born LGA show a higher risk of perinatal morbidity and long-term metabolic complications [9,10,11,12,13].

During the entire reproductive period—but especially in pregnancy—both environmental exposures and lifestyles can affect physiological and pathological conditions in mothers and in future generations [14,15,16]. Maternal diet—which is one of the main determinants of adverse outcomes defined above—plays a key role in ensuring the correct development and growth of newborns [16,17,18,19,20,21,22]. Although many studies evaluated the relationship of single nutrients and foods with pregnancy and neonatal outcomes [23,24,25,26,27,28,29,30,31,32,33,34,35,36], these elements are not consumed alone but in complex dietary patterns. To meet the need of capturing the complex interactions between nutrients, foods and other relevant factors, the research is moving to the development of innovative tools for data collection [37,38,39,40,41,42,43,44,45] and to the analysis of dietary patterns, using both a priori and a posteriori methods [46,47,48,49,50,51,52,53,54,55,56,57,58,59].

Two independent systematic reviews have tried to understand the effects of maternal dietary patterns on birth outcomes [60,61]. There was consistent evidence that certain dietary patterns may affect the risk of PTB [60,61]. Generally, previous studies identified two common dietary patterns among pregnant women: the healthy dietary pattern—characterized by high intakes of plant-based foods and low-fat diary products—was associated with a lower risk of PTB [60,61]; the unhealthy dietary pattern—characterized by high intakes of refined and processed foods and high-fat or high-sugar products—was associated with a higher risk of PTB [60,61]. 

Limited conclusions, instead, can be drawn on the effects of maternal dietary patterns on birth weight [60,61]. A greater adherence to the healthy dietary pattern seemed to be associated with higher birth weight, while the adherence to the unhealthy dietary pattern with a reduced birth weight [60,61]. These findings were partially consistent between studies applying a posteriori data-driven approaches to derive dietary patterns but not between those using a priori index-based dietary patterns [60,61]. Another complication is that birth weight is strictly linked to fetal growth and, therefore, to the duration of gestation and the newborn’s sex [62]. However, in most studies conducted so far, birth weight was not adjusted for gestational age and sex, leading to potentially biased results [60,61]. A previous meta-analysis showed a weak trend towards a reduced risk of SGA in mothers with higher adherence to the healthy dietary pattern, while no association was evident for the risk of LGA [61]. Little research investigated the effects of the unhealthy dietary pattern on birth weight for gestational age [63]. 

Among data-driven methods used in nutritional epidemiology, two multivariate techniques have been widely applied to derive dietary patterns: principal component analysis (PCA) and cluster analysis (i.e., mainly hierarchical and K-means clustering) [47,48,49,53,64,65,66]. To leverage the strengths of these methods, we recently proposed a novel approach combining PCA and clustering for dietary pattern analysis [67]. In the present study, we applied this method to derive dietary patterns among pregnant women from Catania (Italy) and to evaluate the associations with birth weight for gestational age and other pregnancy and neonatal outcomes.

## 2. Materials and Methods

### 2.1. Study Design

The present analysis was conducted on data from the “MAMI-MED” cohort, an ongoing prospective study on mother–child dyads from Catania (Italy), which has been established in December 2020. The general aim of the study is to evaluate the effects of social, environmental, behavioral, and molecular factors on maternal and child health. The MAMI-MED cohort shares the same study protocol and methods used for the “Mamma and Bambino” cohort, established in 2015 [25,26,64,68,69,70,71]. In brief, the study population consists of pregnant women, recruited during the first trimester visit at the “Azienda di Rilievo Nazionale e di Alta Specializzazione (ARNAS) Garibaldi Nesima” (Catania, Italy). The study plan also includes follow-up interviews (i.e., at delivery, and after 12, 24, and 48 months) to collect information about maternal-child health. The study is conducted according to the Declaration of Helsinki after approval by the Ethics committee “Catania 2” with the protocol number 487/CE, 71/2020/CECT2. All women who consent to participate were fully informed about the study purpose and signed their informed consent. In the current analysis, we included mother–child dyads with complete data on socio-demographic characteristics, dietary habits, and birth outcomes.

### 2.2. Data Collection

At recruitment, each woman was administered a structured questionnaire for the collection of socio-demographic characteristics and behaviors. Maternal education was categorized into two levels: low educational level if women had ≤ 8 years of school; high educational level if women had > 8 years of school. Mothers were also classified as unemployed (which included students and housewives) or employed. Regarding the smoking status, they were classified as current or non-current smokers (including ex-smokers). Women were asked about their height and weight before pregnancy at the time of recruitment; this information was then used to calculate and categorize pre-pregnancy BMI according to the WHO criteria [72]. In order to calculate total GWG, the self-reported pre-pregnancy weight was subtracted from the weight achieved at delivery. Using the guidelines of the Institute of Medicine, we defined adequate GWG as a weight gain of 12.5 to 18 kg for underweight, 11.5 to 16 kg for normal weight, 7 to 11.5 kg for overweight, and 5 to 9 kg for obese women [73]. Additional information about gestational duration, birth weight and birth length were collected at delivery. Birth outcomes of interest were PTB (defined as spontaneous delivery before 37 weeks); birth weight for gestational age (defined as SGA, AGA, or LGA according to sex-specific national reference charts [74]).

### 2.3. Dietary Assessment

At recruitment, the assessment included a 95-item semiquantitative Food Frequency Questionnaire (FFQ) referred to the previous 30 days [64,68,71,75,76,77,78]. For each questionnaire item, data on consumption frequency (from “almost never” to “two or more times a day”) and portion size (small, medium, or large) were collected to calculate the daily intakes of foods. Specifically, questionnaire data were converted into daily intakes by multiplying consumption frequencies by the respective portion sizes. Daily intakes of the 95 food items were generally grouped into predefined food categories, but some food items were individually maintained if they constituted a distinct category (e.g., eggs, pizza, coffee or tea, etc.) or if they differentiated a specific dietary habit (e.g., wine, alcoholic drinks, fries, etc.). By doing so, the dietary dataset consisted of 39 food categories reported in the supplementary material. From questionnaire data, the total energy intake was also estimated using the US Department of Agriculture (USDA) Food Composition Database (http://ndb.nal.usda.gov/ (accessed on 1 January 2014)) adapted to Italian foods. Daily dietary intakes were adjusted for total energy intake according to the residual method [79].

### 2.4. Clustering on Principal Components 

To derive distinct dietary patterns, we applied a novel approach defined as clustering on principal components (PCs) that is fully described elsewhere [67]. Briefly, the approach consists of two multivariate techniques to reduce the dimensions of the dietary dataset and to provide the cluster solution. The combination of these methods allows us to take advantage of their strengths, obtaining a better solution than PCA or clustering alone [67]. First, the PCA was applied to reduce the dietary dataset obtained through the FFQ, which was characterized by a set of correlated dietary variables. This common method of data reduction was applied to energy-adjusted daily intakes of the 39 food categories. In this step, we used the varimax rotation on the covariance matrix to improve the interpretability of PC score. The number of PCs to be retained was chosen by visual examination of the scree plot and according to the elbow method. The absolute values of factor loadings were used to determine the contribution of each food category to PCs. For each PC, factor scores were calculated as the sum of products between energy-adjusted intakes and factor loadings. To obtain independent clusters of participants, we next used the hierarchical clustering, an agglomerative method in which data points are progressively merged based on their distance. In our study, the hierarchical clustering was applied to the retained PCs and the distance was measured using Ward’s criterion. The reason for choosing Ward’s Linkage was to work with a multidimensional variance, as already performed with the PCA. Finally, the number of clusters to be considered was chosen according to the silhouette method [67]. In particular, we chose the cluster solution which maximized the silhouette score, a metric based on the intra-cluster distance and separation amongst clusters. 

### 2.5. Statistical Analysis

Statistical analyses were performed using SPSS v. 26.0 (IBM Corp., Armonk, NY, USA). Univariate analysis was conducted to describe the characteristics of the study population, in terms of median and interquartile range (IQR) or frequencies (percentage, %). The Kolmogorov–Smirnov test was used to check the distribution of quantitative variables. Bivariate analysis was conducted using the Mann–Whitney U-test was used for quantitative variables or the Chi-squared test for categorical variables. Z-scores of food and nutrient intakes were compared between clusters using Student’s *t*-test. Multivariable logistic regression analyses were applied to evaluate the association of dietary patterns and birth weight for gestational age (SGA or LGA versus AGA), adjusting age (continuous), pre-pregnancy BMI (continuous), GWG (continuous), educational level (high vs. low-medium), employment status (employed vs. unemployed), primiparity (primiparous vs. non-primiparous), smoking status (smoker vs. non-smoker), and total energy intake (continuous). Results from the logistic regression were reported as Odds Ratios (ORs) and their 95% Confidence Intervals (95%CIs). All statistical tests were two-sided and performed at a significance level α = 0.05.

## 3. Results

### 3.1. Study Population

The study population consisted of 667 women who completed pregnancy and satisfied inclusion criteria. The median age was 31 years (IQR = 7), and 51.1% of women were primiparous. Regarding socioeconomic factors, 24.9% reported a high level of education and 50.7% of women were employed. Almost all of them reported not being a smoker during pregnancy (91%), and they reported a median total energy intake of 1703 kcal (IQR = 508). Before pregnancy, the median BMI was 23.2 kg/m2 (IQR = 5.8), and over half of the women were normal weight (60.4%). According to the GWG (median = 12 kg; IQR = 8), 38.5% of women reported a reduced weight gain, and 28.7% reported an excessive weight gain. The median gestational week at delivery was 39 (IQR = 2), and 94% of births were at term. Regarding birth sizes, the median values were 3.3 kg (IQR = 0.6) for birth weight and 50 cm (IQR = 2) for birth length. As a result, 81.9% of newborns were AGA, 7.1% were SGA, and 11.0% were LGA.

### 3.2. Derivation of Clusters Reflecting Distinct Dietary Patterns

**Figure 1:**
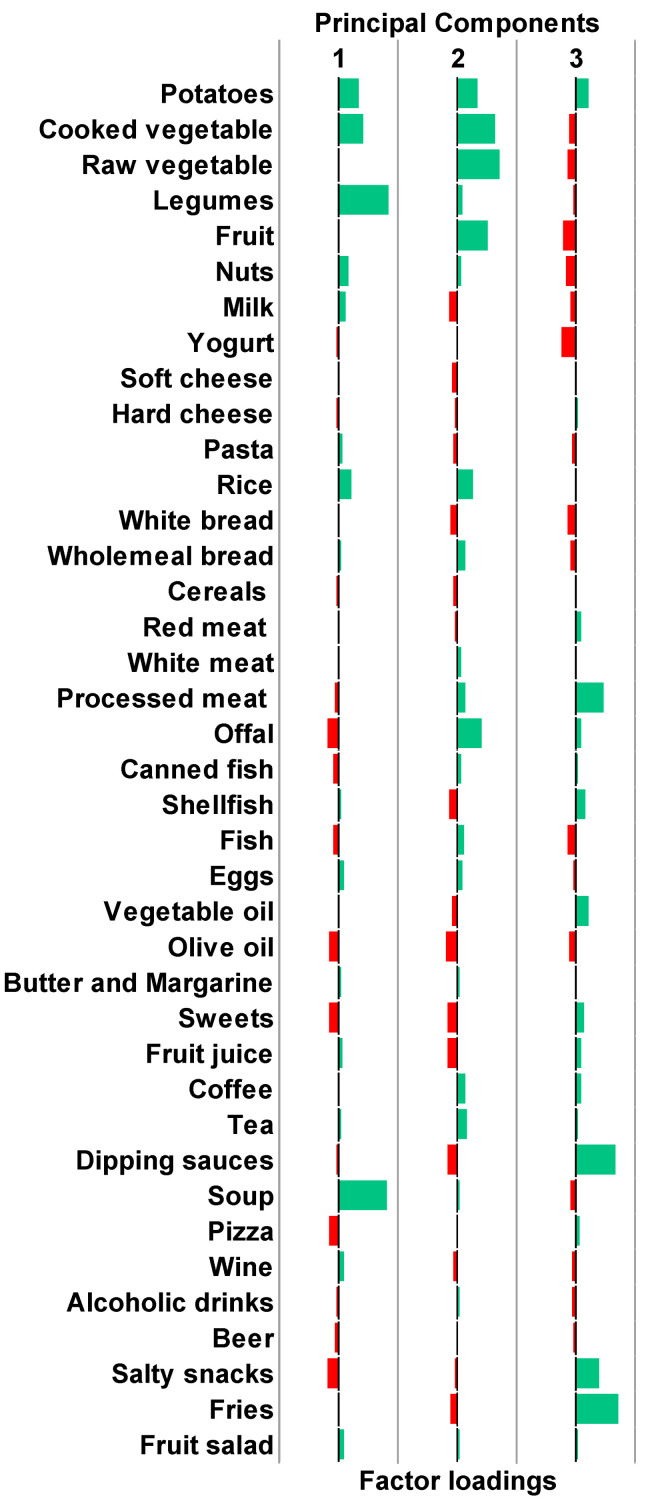
Factor loadings of the three main principal components. Green and red bars represent food items that positively or negatively characterized the principal components.

**Figure 2:**
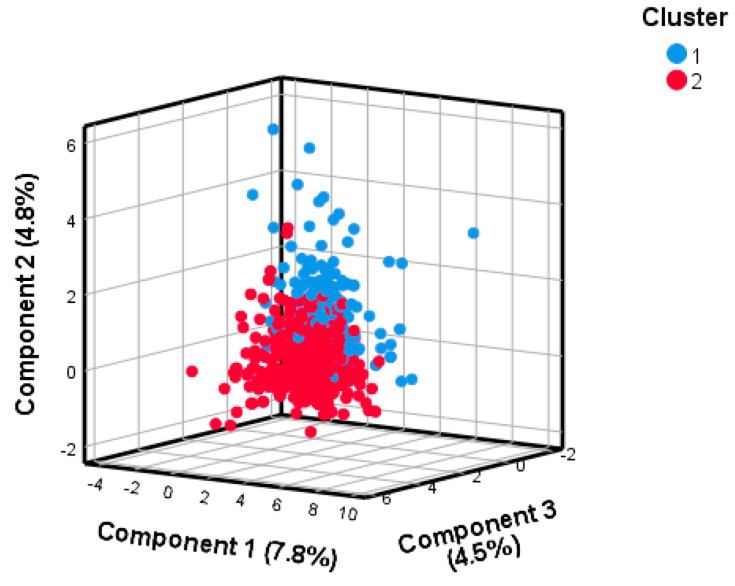
Distribution of participants by principal components and clusters.

**Figure 3:**
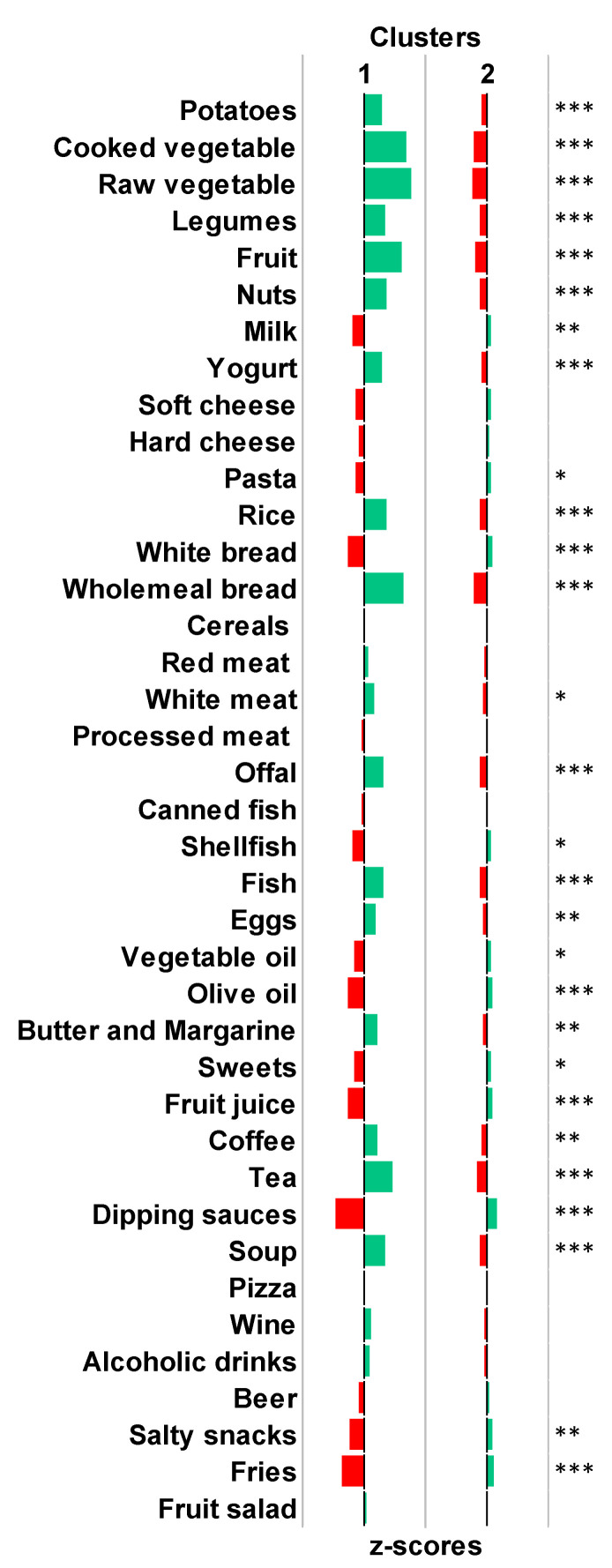
Comparison of dietary intakes between clusters. Green bars represent food items that positively characterized the cluster. Red bars represent food items that negatively characterized the cluster. According to Student’s *t*-test, results are reported as *** for *p*-values < 0.001; ** for *p*-values < 0.01; * for *p*-values < 0.05.

After checking sampling adequacy and sphericity assumptions (KMO = 0.741 and *p*-value for the Bartlett’s test < 0.001), the PCA was applied to standardized and energy-adjusted dietary data. The PCA produced 16 PCs with eigenvalue greater than 1. Through inspection of the scree plot and in accordance with the elbow method (Figure S1), we selected the first three PCs which together explained 17.2% of the total variance. Figure 1 depicts the factor loadings associated with each PC: PC1 was mainly characterized by the intake of potatoes, cooked vegetables, legumes, and soup; PC2 was mainly characterized by the intake of potatoes, cooked and raw vegetables, fruit, and offal; PC3 was mainly characterized by the intake of processed meat, dipping sauces, salty snacks, and fries. Next, we applied the hierarchical clustering on PC1, PC2, and PC3, obtaining the dendrogram shown in Figure S2. We also calculated silhouette scores for different clustering solutions and chose the one characterized by two clusters (Figure S3). Figure 2 shows the distribution of participants according to the three main PCs and the clustering classification, while Figure 3 compares the average z-scores for each dietary category between clusters. Participants in cluster 1 (*n* = 158) were characterized by higher intake of potatoes, cooked and raw vegetables, legumes, fruits, nuts, yogurt, rice, wholemeal bread, white meat, offal, fish, eggs, butter and margarine, coffee, tea, and soup. Those in cluster 2 (*n* = 509) were characterized by higher intake of milk, pasta, white bread, shellfish, vegetable and olive oils, sweets, fruit juices, dipping sauces, salty snacks, and fries. Regarding nutrients, participants in cluster 1 were characterized by higher intakes of folate, magnesium, and vitamins A, B6, and C, while those belonging to cluster 2 had higher intakes of saturated and unsaturated fatty acids, calcium, and vitamin B1 (Figure S4). 

### 3.3. Differences in Maternal Characteristics and Birth Outcomes according to Dietary Patterns

**Figure 4:**
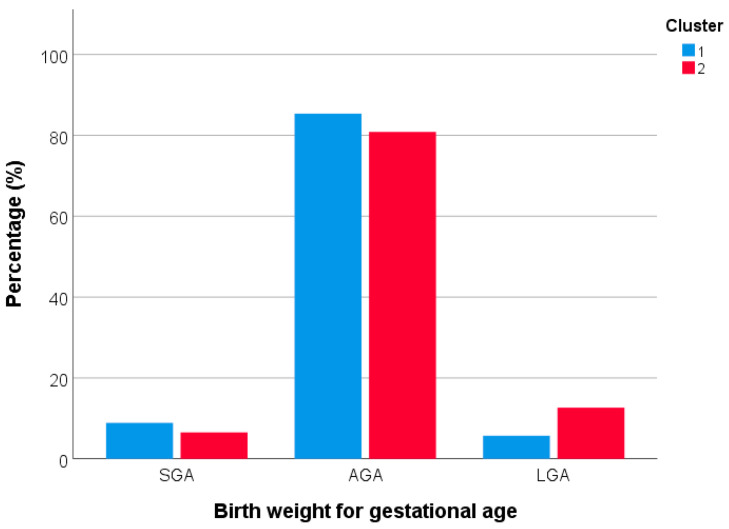
Distribution of birth weight for gestational age by clusters.

Subsequently, we compared the characteristics of women based on their membership in one of the two clusters. Women belonging to cluster 2 were younger (*p* < 0.001) and had a lower level of education (*p* = 0.018) than those belonging to cluster 1. Furthermore, they reported a higher total energy intake (*p* < 0.001). No other statistically significant differences were evident between the clusters. Likewise, there were no differences between the clusters regarding birth outcomes, including gestational week at delivery, proportion of preterm birth, birth weight, and birth length (Table 1). However, there was a significant difference in terms of birth weight for gestational age. Specifically, we found a higher proportion of LGA among those born from women belonging to cluster 2. On the other hand, there were higher proportions of SGA and AGA among those born to women belonging to cluster 1 (*p* = 0.030; Figure 4).

### 3.4. Factors Associated with Birth Weight for Gestational Age

Finally, we evaluated which were the main factors associated with birth weight for gestational age (Table 2). Regarding SGA, the main associated factors were employment status and primiparity. Newborns from employed women had lower odds of SGA than those who were unemployed (OR = 0.359; 95%CI = 0.168–0.769; *p* = 0.008). Conversely, primiparous women had higher odds of SGA than women who have already had one or more pregnancies (OR = 2.681; 95%CI = 1.293–5.558; *p* = 0.008). Regarding LGA, the main associated factors were cluster membership and pre-pregnancy BMI. Specifically, women belonging to cluster 2 had higher odds of LGA than those belonging to cluster 1 (OR = 2.213; 95%CI = 1.047–4.679; *p* = 0.038). Moreover, the odds of LGA increased by nearly 11% for each one-unit increase in pregestational BMI (OR = 1.107; 95%CI = 1.053–1.163; *p* < 0.001).

## 4. Discussion

To our knowledge, this is the first study applying the clustering on PCs to derive dietary patterns during pregnancy and to evaluate their associations with pregnancy and birth outcomes, with a particular focus on the effect on birth weight for gestational age. By applying this method, we first obtained two clusters reflecting dietary patterns that were popular among pregnant women. The identified dietary patterns were similar to those derived in previous studies on similar populations from the same region [48,49,64,77,80,81]. The first dietary pattern can be considered healthier, because it was mainly characterized by plant-based foods (e.g., potatoes, cooked and raw vegetables, legumes, soup, fruits, nuts, rice, wholemeal bread), fish and white meat, eggs, butter and margarine, coffee, and tea. The second resembled a western diet because it consisted mainly of junk foods (sweets, dips, salty snacks, and fries), pasta, white bread, milk, vegetables, and olive oils. Regarding nutrient content, the healthy dietary pattern was rich in folate, magnesium, and vitamins A, B6, and C, while the western diet was characterized by high intake of calories, fatty acids, calcium, and vitamin B1. 

From the comparison of maternal characteristics between dietary patterns, it was evident that younger and less educated women adhered more to the western diet. These findings were in line with previous evidence showing that younger people with lower level of education are moving away from traditional healthy habits and instead adopting western behaviors [48,54,55,78,81,82,83,84]. Consistent with research conducted in other European countries, younger Italian women had poorer diet quality, which suggests that health consciousness tends to improve as people age [85,86]. Furthermore, individuals with higher levels of education tend to possess greater knowledge about the benefits and risks associated with their dietary choices [87], making education the most significant sociodemographic factor influencing healthy decision-making [88].

In our study, there were no differences between dietary patterns in terms of gestational week at delivery and PTB. This finding differed from those obtained by previous meta-analyses and interventional studies [60,61,89], although it should be noted that PTB was quite rare in our study population. Instead, we found a significant association of dietary patterns with birth weight for gestational age. This was already evident from the bivariate analysis, which showed a higher proportion of LGA births among women who adhered to the western dietary pattern and higher proportions of SGA and AGA among those who followed the healthy profile. However, in the multivariable analysis, only the relationship with LGA remained significant: in particular, the odds of LGA were 2.2 times higher in women who adhered to the western dietary pattern than in those who adhered to the healthy profile. This evidence improves the current understanding of the relationship between unhealthy dietary patterns and birth weight for gestational age, as previous systematic reviews have shown that studies in this area are currently limited and inconsistent [60,61]. It is worth noting that this association was adjusted for the effect of covariates, including total energy intake and GWG. Although a maternal diet that increases caloric intake and weight gain might affect LGA risk, our results suggest other mechanisms are also involved. Another factor associated with LGA was pregestational BMI, with a higher likelihood of LGA as BMI increased. It is in fact well established that higher maternal BMI is associated with a greater risk of delivering a high birth weight newborn, while a lower maternal BMI is associated with a greater risk of delivering a LBW newborn [5]. Though we recognize that scientific evidence is not yet sufficient to make a definitive recommendation on this issue, our findings are important because they provide the foundation for future studies evaluating maternal diet’s impact on birth weight. 

The absence of the relationship with the risk of SGA was not surprising. As Raghavan and colleagues reported in their previous systematic review, only half of the studies found an association between dietary patterns and birth weight outcomes [60]. Furthermore, there was limited consistency in dietary patterns and direction of effect among studies that showed significant associations [60]. Another limitation was that half of these studies did not adjust birth weight for gestational age, making it impossible to discriminate whether the effect was on birth weight or gestational duration [60]. Similar findings were obtained by Chia and colleagues, who included in their systematic review and meta-analysis 18 studies examining the association of dietary patterns with SGA, IUGR, or LBW [61]. In their study, the adherence to healthy dietary patterns was associated with a non-significant slight reduction in the risk of SGA, IUGR, or LBW [61]. Therefore, other factors mainly contribute to the risk of SGA birth. In our multivariable analysis, the only factors associated with SGA were primiparity and employment status. If for the first the results confirmed the notion that primiparous women are at higher risk of SGA and other complications [90], for the second it is necessary to discuss it in detail. In our study, employed women had lower odds of giving birth to SGA newborns than their unemployed counterpart. These results were consistent with some previous studies showing a positive effect of maternal employment on the risk of SGA, as well as on BMI in children [91,92]. However, in order to better understand the relationship, it is important to investigate potential confounding and mediating factors in the future, such as the type of employment, working hours, income, daily activities, and other relevant factors.

The main strength of our study is the application of an innovative method for analyzing dietary patterns during pregnancy. This approach overcomes the limitations of PCA (which does not provide distinct dietary patterns but scores for different components) and provides a better solution than simple clustering [67]. In addition, the analyses were conducted on a large dataset, consisting of more than 600 records and 50 variables. By applying multivariable models, we are confident that the association between the adherence to a western diet and the risk of LGA did not depend on potential confounders, such as total energy intake, pre-pregnancy BMI, and weight gain during pregnancy. However, it is not possible to completely rule out the possibility that other unmeasured factors may have an impact on the observed relationship. Our study has additional limitations to be considered. Firstly, the low percentages of adverse outcomes—and in particular of LGA and SGA—may have influenced the statistical power of our analyses. Due to this limitation, it was not possible to examine the impact on metabolic risk and gestational diabetes during pregnancy as well. To do that, the perspective is to design ad hoc studies focusing on subgroups of women at higher risk of adverse outcomes. A further limitation is the use of FFQ as a tool for dietary assessment. Although it represents the gold standard in nutritional epidemiology, it is not free from potential biases. Moreover, in the near future, it could be interesting to evaluate the effect of dietary patterns and nutrient content not only in the early pregnancy but also in close proximity to childbirth. 

Despite these limitations, the present study is the first to highlight a relationship between the adherence to an unhealthy dietary pattern and the likelihood of giving birth to an LGA newborn. This evidence adds to the current knowledge about the effects of diet on birth weight, which, however, remains limited and controversial. Although far from drawing definitive recommendations based on evidence, these findings are important to guide future research in this field. In particular, our work proposes an alternative method for analyzing nutritional profiles, which could be used in future studies during pregnancy and throughout the periconceptional period. 

## Figures and Tables

**Table 1:** Characteristics of the study population by clusters reflecting dietary patterns.

Characteristics	Cluster 1 (*n* = 158)	Cluster 2 (*n* = 509)	*p*-Value ^a^
Age (years) ^b^	32.0 (5.0)	30.0 (7.0)	<0.001
High educational level	29.7%	23.4%	0.018
Employed	55.1%	49.3%	0.207
Non-smoker	94.9%	89.8%	0.055
Primiparous	46.5%	52.5%	0.191
Total energy intake (kcal/day) ^b^	1567 (486)	1749 (503)	<0.001
Pre-pregnancy BMI (kg/m^2^) ^b^	23.5 (5.4)	23.2 (5.9)	0.373
Pre-pregnancy BMI classification			
Underweight	5.7%	5.3%	0.965
Normal weight	58.6%	60.9%	
Overweight	22.3%	21.2%	
Obese	13.4%	12.6%	
GWG (kg) ^b^	11.0 (8.3)	12.0 (8.0)	0.272
GWG classification			
Reduced	42.9%	37.2%	0.289
Adequate	27.9%	34.3%	
Excessive	29.2%	28.5%	
Gestational week at delivery (weeks) ^b^	39.0 (2.0)	39.0 (2.0)	0.489
Preterm birth	8.3%	5.3%	0.174
Birth weight (kg) ^b^	3.2 (0.6)	3.3 (0.6)	0.171
Birth length (cm) ^b^	50.0 (2.0)	50.0 (2.0)	0.233

^a^ *p*-values are obtained through the Chi-squared test for qualitative variables and the Mann–Whitney U-test for quantitative variables. ^b^ Data are reported as median (IQR). Abbreviations: BMI, Body Mass Index; GWG, Gestational Weight Gain.

**Table 2:** Factors associated with birth weight for gestational age.

Characteristics	SGA		LGA	
	OR (95%CI)	*p*-Value	OR (95%CI)	*p*-Value
Cluster 2 vs. Cluster 1	0.537 (0.262–1.104)	0.091	2.213 (1.047–4.679)	0.038
Age (continuous)	0.965 (0.894–1.041)	0.356	0.955 (0.899–1.014)	0.132
Pre-pregnancy BMI (continuous)	1.003 (0.939–1.071)	0.934	1.107 (1.053–1.163)	<0.001
GWG (continuous)	0.966 (0.928–1.005)	0.089	1.030 (0.997–1.064)	0.075
High educational level	1.060 (0.617–1.821)	0.834	1.154 (0.754–1.767)	0.509
Employed	0.359 (0.168–0.769)	0.008	0.745 (0.414–1.341)	0.327
Primiparous	2.681 (1.293–5.558)	0.008	0.980 (0.563–1.704)	0.942
Smoker	1.841 (0.697–4.865)	0.218	0.352 (0.102–1.214)	0.098
Total energy intake (continuous)	1.000 (1.000–1.001)	0.207	1.000 (1.000–1.001)	0.474

Results are obtained by applying logistic regression models including dietary patterns (cluster 2 vs. Cluster 1), age (continuous), pre-pregnancy BMI (continuous), GWG (continuous), educational level (high vs. low-medium), employment status (employed vs. unemployed), primiparity (primiparous vs. non-primiparous), smoking status (smoker vs. non-smoker), and total energy intake (continuous).

## Data Availability

The data presented in this study are available on request from the corresponding author.

## Supplementary Materials

The following supporting information can be downloaded at: https://www.mdpi.com/article/10.3390/nu15081922/s1, Table S1: List of 39 food categories obtained from the Food Frequency Questionnaire; Figure S1: Scree plot of Principal Component Analysis; Figure S2: Dendrogram of the hierarchical clustering; Figure S3: Silhouette scores for different cluster solutions. Figure S4: Comparison of nutrients intakes between clusters.
