# Supplementary material for: The Effect of Maternal Dietary Patterns on Birth Weight for Gestational Age: Findings from the MAMI-MED Cohort
Source: Nutrients. 2023 Apr 16;15(8):1922. doi: 10.3390/nu15081922 (PMC10147093; doi:10.3390/nu15081922)

Supplementary Materials

Table S1. List of 39 food categories obtained from the Food Frequency Questionnaire

95 FFQ Food Items	39 Food Groups
Spirits and other alcoholic drinks	Alcoholic drinks
Beer	Beer
Butter and margarine	Butter and Margarine
Canned fish	Canned fish
Cereals	Cereals
Coffee	Coffee
Cooked vegetables	Cooked vegetables
Spinach	
Carrots	
Peppers	
Aubergine	
Cauliflower, broccoli	
Zucchini, pumpkin	
Artichoke	
Mushroom	
Ketchup	Dipping sauces
Mayonnaise	
Eggs	Eggs
Fish (high in fat)	Fish
Fish (medium in fat)	
Fish (low in fat)	
Fries	Fries
Apple	Fruit
Pear	
Banana	
Peach, nectarine, prune	
Apricot	
Orange, mandarin	
Strawberries	
Melon, mango	
Watermelon	
White grape	
Red grape	
Pineapple	
Cherries	
Kiwi	
Multivitamin juice	Fruit juice
Pineapple juice	

Orange juice	
Pear juice	
Peach juice	
Fruit salad	Fruit salad
Hard cheeses	Hard cheese
Legumes	Legumes
Green bean	
Peas	
Whole milk	Milk
Semi-skimmed milk	
Skimmed milk	
Nuts	Nuts
Offal	Offal
Olive oil	Olive oil
Pasta	Pasta
Pizza	Pizza
Boiled potatoes	Potatoes
Salami	Processed meat
Ham (cooked or uncooked)	
Bresaola	
Fesa, chicken breast	
Wurstel pork	
Wurstel chicken	
Mortadella	
Green salad	Raw vegetable
Fresh tomatoes	
Fennel	
Beef, veal, lamb meat	Red meat
Pork meat	
Horse meat	
Rice	Rice
Popcorn, pretzels	Salty snacks
Wrapped potato chips	
Shellfish	Shellfish
Soft cheeses	Soft cheese
Soup	Soup
Cake, pastries	Sweets
Ice cream	
Pastry cream, pudding	
Chocolate, snack chocolate	
Candy	
Jam, marmalade	

Tea	Tea
Vegetable oil	Vegetable oil
Brioches	White bread
Cookies	
Bread	
Rusks, crackers	
White meat	White meat
Wholemeal biscuits	Wholemeal bread
Wholemeal bread	
Rusks, wholemeal crackers	
Red wine	Wine
Rosè wine, white wine	
Full fat yoghurt	Yoghurt
Low fat yoghurt	
Water	Not applicable

Figure S1. Scree plot of Principal Component Analysis

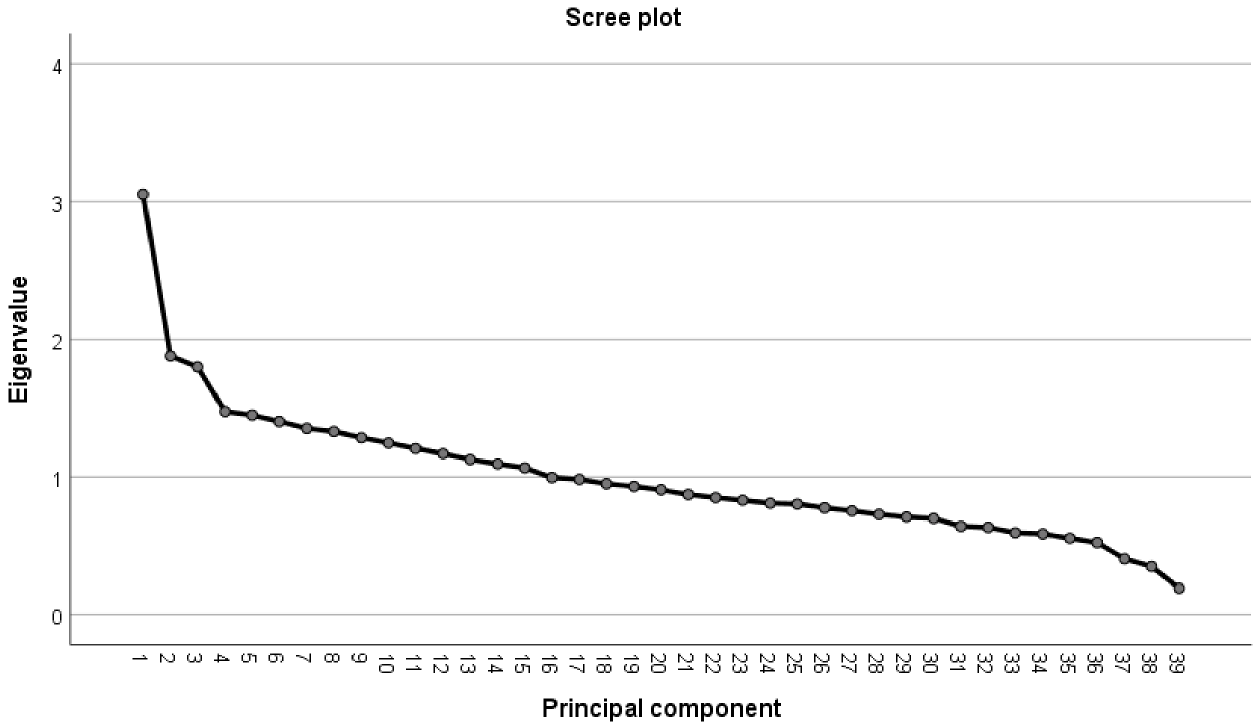

Figure S2. Dendrogram of the hierarchical clustering

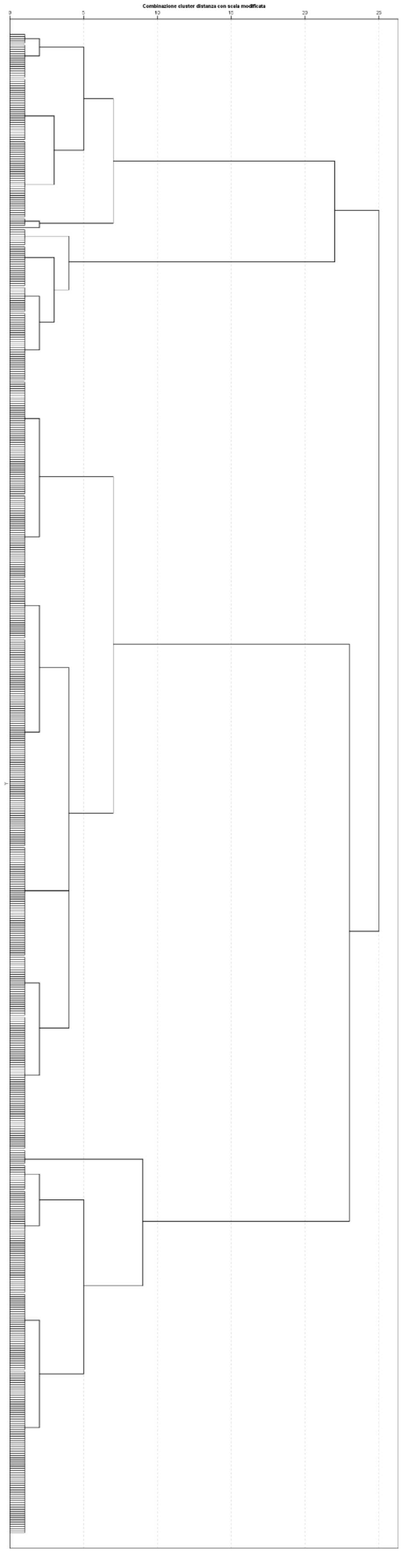

Figure S3. Silhouette scores for different cluster solutions

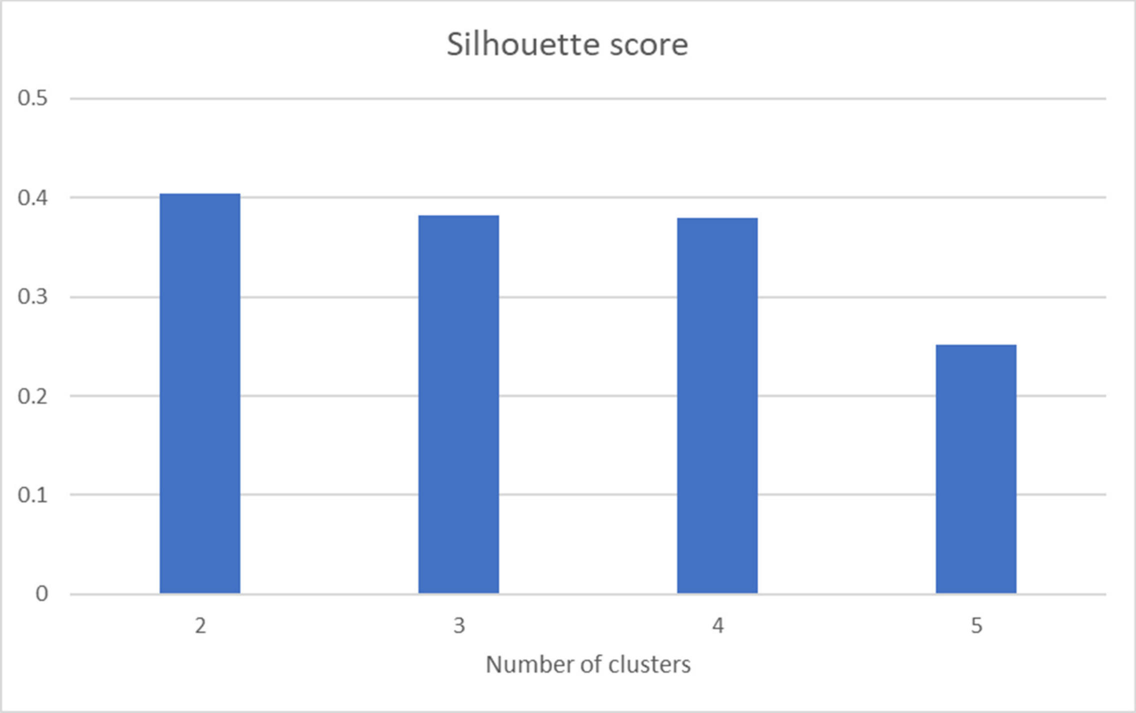

Figure S4. Comparison of nutrients intakes between clusters.
Green bars represent nutrient intakes that positively characterize the cluster. Red bars represent nutrient intakes that negatively characterize the cluster. According to the Student's t-test, results are reported as *** for p-values <0.001; ** for p-values <0.01; * for p-values <0.05.

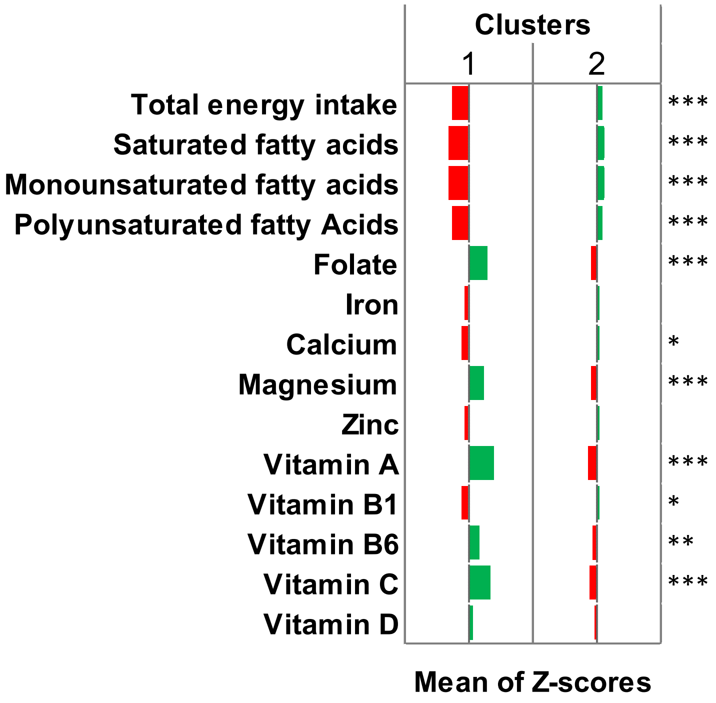

Supplement: Supplementary file 1 [file nutrients-15-01922-s001.zip › nutrients-2279572-supplementary.pdf]
